# Modulating One-Carbon Metabolism with B-Vitamins to Protect the Retinal Barrier and Prevent Retinal Degeneration †

**DOI:** 10.3390/nu18020236

**Published:** 2026-01-12

**Authors:** Hossameldin Abouhish, Lamiaa Shalaby, Omar Elzayat, Neelesh Peddireddy, Amany Tawfik

**Affiliations:** 1 Eye Research Institute, Oakland University, Rochester, MI 48309, USA; habouhish@oakland.edu (H.A.); shalaby@oakland.edu (L.S.); omarelzayat@oakland.edu (O.E.); 2 Department of Clinical Pharmacology, Faculty of Medicine, Mansoura University, Mansoura 35516, Egypt; 3 Eye Research Center, Oakland University William Beaumont School of Medicine, Rochester, MI 48309, USA; npeddireddy@oakland.edu

**Keywords:** B-vitamins, homocysteine, blood–retinal barrier, age-related macular degeneration, diabetic retinopathy

## Abstract

Background/Objectives: Vitamin B_12_ deficiency is increasingly recognized as a contributor in both vascular and neurodegenerative aging-related disorders. Its deficiency disrupts one-carbon metabolism, leading to impaired homocysteine (Hcy) cycling. Elevated Hcy is a well-established risk factor for vascular dysfunction. Previously, we established that elevated Hcy contributes to aging retinal diseases and plays a central role in blood retinal barrier (BRB) dysfunction. Building on this foundation, the present study examines how B-vitamin deficiency disrupts one-carbon metabolism and whether restoring these vitamins can serve as a preventive or therapeutic strategy. Since B-vitamins (B_6_, B_9_, and B_12_) are crucial cofactors in the metabolism of Hcy, we investigated how dietary changes in these vitamins affect serum Hcy levels and retinal vascular integrity in mice. Methods: C57BL/6- Wild-type (WT) and *cbs^+/−^* mice (Cystathionine Beta-Synthase heterozygotes, common mouse model for elevated Hcy) were fed specially formulated diets, which contained different levels of B-vitamins (normal, deficient (B-Vit (−)) or enriched (B-Vit (+)). Initially, two groups of mice were placed on either a normal or a deficient diet. After 12–16 weeks, the success of the diet regimes was confirmed by observing serum B_12_ deficiency in the B-Vit (−) group, along with elevated Hcy levels. Subsequently, a subgroup of the B-Vit (−) mice was switched to an enriched diet. The BRB integrity was evaluated in living mice using fluorescein angiography (FA), optical coherence tomography (OCT), and in the perfused mice retinas with Western blot analysis of leaked retinal albumin and tight junction proteins (occludin and ZO-1) levels. Results: The B-vitamin deficiency caused significant drop in serum vitamin B_12_ and an increase in plasma Hcy, leading to vascular leakage, altered retinal thickness, choroidal neovascular changes, increased retinal albumin leak, and decreased tight junction protein expression, indicating BRB disruption, which was restored with B-vitamin supplementation. Conclusions: a long-term deficiency of vitamins B_6_, B_9_, and B_12_ can lead to disruptions in the BRB. However, supplementation with these B-vitamins has the potential to reverse these effects and help maintain the integrity of BRB. This under-score the significance of one-carbon metabolism for retinal health and suggests that ensuring adequate levels of B-vitamins may aid in preventing aging retinal diseases with BRB disruption such as diabetic retinopathy and age-related macular degeneration.

## 1. Introduction

Homocysteine (Hcy) is an amino acid that is produced during the metabolism of methionine in the one-carbon cycle. Its levels are closely regulated by vitamins B_6_ (pyridoxine), B_9_ (folate), and B_12_ (cobalamin), which act as cofactors in the remethylation and transsulfuration pathways that help dispose of Hcy [1,2,3]. Deficiencies or imbalances in any of the B-vitamins can lead to the elevation of Hcy levels.

Previous research has associated B-vitamins with altered Hcy levels and their subsequent effects on health. Ortiz-Salguero et al. [4] reviewed evidence suggesting that B-vitamins supplementation may exert protective effects, especially among individuals with metabolic or vascular disorders. Similarly, Nieraad et al. [5] reported that animals consuming diets deficient in vitamins B_6_, B_12_, or folate exhibited elevated Hcy levels. Elevated Hcy, in turn, has been implicated not only in cardiovascular disease [6,7,8] but also in microvascular and neurovascular dysfunction, including retinal involvement [9].

The retina has a specialized protective system called the blood retinal barrier (BRB), which regulates the exchange of molecules between the bloodstream and neural retina, thus maintaining retinal homeostasis and ensuring proper neuronal function [10]. The disruption of the BRB integrity is a key characteristic of major retinal vascular diseases, including Diabetic Retinopathy (DR), that is associated with disruption of the inner BRB and Age-related Macular Degeneration (AMD), that is associated with disruption of the outer BRB [11,12]. Research has demonstrated that elevated levels of Hcy are linked to retinal vascular occlusive diseases in humans. For example, patients with retinal vein occlusion typically exhibit higher levels of Hcy [13].

Our laboratory has extensively investigated the harmful effects of elevated Hcy on the integrity of the BRB [14,15,16], using various mouse models. These include genetic models with cystathionine β-synthase (CBS) deficiency and pharmacologically induced models created through Hcy administration intraocular [17]. We have explored the underlying mechanisms, which involve oxidative stress resulting from the generation of reactive oxygen species (ROS) [18], mitochondrial dysfunction [19], endoplasmic reticulum stress [20], and the activation of vascular N-methyl-D-aspartate receptors (NMDARs) [21]. Additionally, as indicated in our previous study, in vitro experiments with human retinal endothelial and retinal epithelial pigmented cells, main components of inner and outer BRB, have shown that exposure to Hcy leads to the disruption of tight junctions, increased permeability, and oxidative stress. These findings suggest that elevated Hcy may have direct harmful effects on the structure and function of the BRB [17,18].

In age-related disorders such as Alzheimer’s disease, Parkinson’s disease, and age-associated macular degeneration, impaired methylation and elevated Hcy have been linked to neuronal apoptosis, inflammation, and compromised microvascular perfusion [6,8,9]. Nutritional modulation through adequate dietary intake or supplementation of B- vitamins can therefore help preserve vascular integrity, reduce neuroinflammatory processes, and potentially slow cognitive and retinal decline. This underscores the importance of optimizing B-vitamin levels as a cost-effective and accessible strategy to slow the progression of age-related vascular and neurodegenerative diseases.

In rodent models, dietary manipulations that modify levels of methionine, folate, and vitamins B_6_ and B_12_ are used to induce elevated Hcy, providing a platform to investigate its vascular effects. For example, a recent systematic review on cognitive dysfunction reported that deficiencies in vitamins B_6_, B_12_, or folate consistently increase plasma Hcy levels in mice and rats [5]. Similarly, an in vivo study examining Hcy-induced cognitive impairment established an elevated Hcy level by feeding mice a specialized diet [22]. Adjusting the dietary concentrations of vitamins B_6_, B_9_, and B_12_ allows for controlled exploration of Hcy-driven vascular changes.

In this study, we used a specially formulated diet with varying levels of vitamins B_6_, B_9_, and B_12_ to induce elevated Hcy in mice and examined its effects on the BRB. Building on evidence that dietary B-vitamin deficiency causes elevated Hcy levels and vascular dysfunction, we assessed tight junction protein expression, capillary permeability, and retinal vascular morphology. Our findings link nutritional modulation of one-carbon metabolism to BRB integrity, suggesting that elevated Hcy contributes to microvascular barrier breakdown. This model provides insight into how B-vitamin deficiencies may promote retinal vascular diseases such as DR and AMD in humans.

## 2. Materials and Methods

### 2.1. Animals and Treatments

C57BL/6 wild-type (WT) and cystathionine β-synthase heterozygous (*cbs^+/−^*) mice (Jackson Laboratories, Bar Harbor, ME, USA) were used in this study. Mice were maintained under controlled environmental conditions with a 12 h light/12 h dark cycle at a constant temperature of 22–24 °C, with ad libitum access to food and water. To prevent light-induced retinal damage, illumination at the bottom of the cages was maintained at 1.5 foot-candles. All experimental procedures were approved by the Institutional Animal Care and Use Committee (IACUC) of Oakland University (Protocol # 2022-1160) and adhered to the ARVO Statement for the Use of Animals in Ophthalmic and Vision Research, as well as the Public Health Service Guide for the Care and Use of Laboratory Animals (Department of Health, Education, and Welfare publication, NIH 80-23).

**Figure 1:**
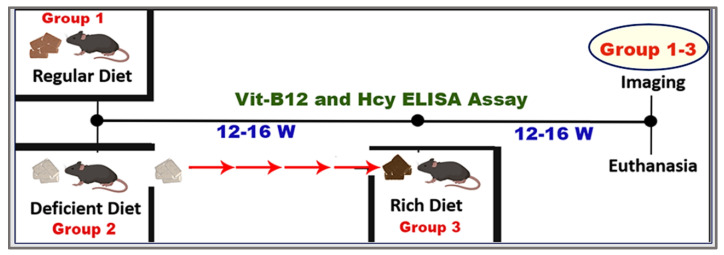
**Experimental timeline for dietary modulation and outcome assessments in mice.** Mice were initially placed on either a regular diet or a B-vitamin deficient diet. After 12–16 weeks on the assigned diet, blood samples were collected for vitamin B_12_ and Hcy assays. A subgroup of animals on the deficient diet was subsequently switched to a B vitamin-rich diet. 16 weeks later, mice underwent imaging assessments, followed by euthanasia for tissue collection and further analysis.

Both WT and *cbs^+/−^* Mice were fed a modified AIN-93G diet (Bio-Serv, Inc., Flemington, NJ, USA) formulated with varying levels of vitamins B_6_, B_9_ (folic acid), and B_12_. Dietary vitamin concentrations were calculated based on an average mouse body weight of 25 g and a daily food intake of approximately 5 g. Using the target doses expressed as mg/kg body weight per day, the corresponding dietary concentrations were determined as follows: folic acid, 300 mg/kg diet (equivalent to 60 mg/kg body weight/day); vitamin B_6_, 250 mg/kg diet (equivalent to 50 mg/kg body weight/day); and vitamin B_12_, 5 mg/kg diet (equivalent to 1.0 mg/kg body weight/day). Three dietary conditions were created: (1) a regular diet (catalog #S3156) containing standard B-vitamin levels, (2) a vitamin-deficient diet (B-Vit (−); catalog #F10342) with reduced B_6_, B_9_, and B_12_, and (3) a vitamin-enriched diet (B-Vit (+); catalog #F10341) with elevated B-vitamin content. Initially, mice were assigned to either the regular or deficient diet groups. 12–16 weeks later, blood samples were collected to confirm B-vitamin deficiency and elevated Hcy levels via serum vitamin B_12_ ELISA and Hcy assays. A subset of B-Vit (−) mice was then transitioned to the enriched diet for an additional 16 weeks to evaluate recovery (Figure 1). The entire experimental procedure, including dietary interventions, monitoring, and group transitions, was conducted in triplicate to ensure reproducibility; each group contained 8–10 mice, and each mouse was considered an experimental unit. Table 1 displays group distribution, study design, and experiments conducted. All animal procedures followed institutional and ethical guidelines for the care and use of laboratory animals.

### 2.2. Fluorescent Angiography (FA) and Optical Coherence Tomography (OCT)

Retinal imaging was performed in live mice to assess vascular integrity, retinal morphology, and thickness using fluorescein angiography (FA) and Optical Coherence Tomography (OCT), conducted with the Phoenix Micron IV system (Phoenix Research Laboratories, Pleasanton, CA, USA). Mice were anesthetized with 1.5–2% isoflurane in oxygen, delivered via a nose cone to ensure stable and consistent sedation throughout the procedure. Before imaging, a 1% tropicamide ophthalmic solution was applied topically to induce pupil dilation and enable clear visualization of the retina.

For FA, mice were injected intraperitoneally with 10–20 μL of 10% fluorescein sodium solution (Apollo Ophthalmics, Newport Beach, CA, USA). Sequential angiographic images were captured at various time points post-injection to monitor dye perfusion and vascular leakage. Quantitative analysis of fluorescein intensity and vascular leakage was performed using ImageJ software (version 1.54g; National Institutes of Health, Bethesda, MD, USA). OCT Imaging was performed using the same Phoenix Micron IV OCT system (Phoenix Research Laboratories, Pleasanton, CA, USA). To maintain corneal hydration and optical clarity during imaging, Goniovisc 2.5% hypromellose solution (Sigma Pharmaceuticals, LLC, Monticello, IA, USA) was applied to the eye surface. Quantitative measurements of retinal layer thickness were obtained using the OCT system’s proprietary software, version 2.1.7237.

**Figure 2:**
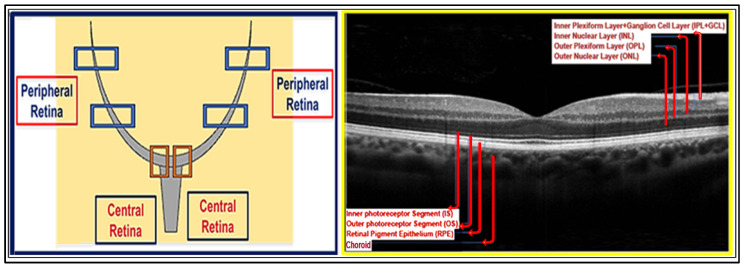
**Retinal regions and layer segmentation used for OCT analysis.** Left panel: Schematic representation of the mouse retina illustrating the predefined regions of interest used for quantitative analysis. Central retina measurements were obtained adjacent to the optic nerve head (Orange boxes), while peripheral retina measurements were collected from equidistant superior and inferior locations (blue boxes). Right panel: Representative OCT scan showing retinal layer segmentation. Quantified layers included the inner plexiform layer, ganglion cell layer complex (IPL + GCL), inner nuclear layer (INL), outer plexiform layer (OPL), and outer nuclear layer (ONL), as well as the photoreceptor inner segment (IS), outer segment (OS), retinal pigment epithelium (RPE), and choroid. These regions and layers were consistently applied across all experimental groups.

To avoid pseudoreplication, the animal was treated as the independent experimental unit for all analyses. Both eyes were imaged at the time points as indicated in Table 1; however, values were first averaged per eye across predefined regions of interest (ROIs) and then averaged per animal before statistical testing. Central and peripheral ROIs were sampled using a standardized, predefined approach that was applied identically across all groups, as shown in Figure 2. All image quantifications were performed by an investigator blinded to experimental group assignment until completion of the analysis.

### 2.3. Western Blot Analysis

After perfusion and euthanizing the experimental mice, their eyes were promptly enucleated to isolate the retinas, which were carefully dissected and immediately snap-frozen in liquid nitrogen to preserve protein integrity, then stored at -80 °C until use. To extract protein, the frozen retinal tissues were homogenized in ice-cold RIPA buffer (Thermo Fisher Scientific, Waltham, MA, USA) containing a protease and phosphatase inhibitor cocktail (Thermo Fisher) to prevent degradation and dephosphorylation of proteins.

Concentrations of protein in the lysates were quantified according to the manufacturer’s protocol for the BCA Protein Assay (Pierce™ BCA Protein Assay Kit, Thermo Fisher Scientific). Laemmli sample buffer was added to the protein lysate, and then the mixture was boiled at 95 °C for 5 min. Equal amounts of protein from each sample were separated using SDS-PAGE with a gradient gel (4% to 20%, Pierce, Rockford, IL, USA). After the separation, Proteins were transferred onto polyvinylidene difluoride (PVDF) membranes (MilliporeSigma, Burlington, MA, USA) using a wet or semi-dry transfer system. Following transfer, blocking of the membranes was performed using 5% non-fat dry milk dissolved in Tris-buffered saline with 0.1% Tween-20 (TBST) for 1 h at room temperature. Blocked membranes were incubated overnight at 4 °C with primary antibodies specific to target proteins, including anti-Albumin (Cell Signaling Technology, Danvers, MA, USA, catalog # 4929), anti-Occludin (Invitrogen, Waltham, MA, USA, catalog # 71-1500), and anti-ZO-1 (Thermo Fisher, catalog # 40-2200). After overnight incubation with primary antibodies, membranes were washed thoroughly with TBST and kept at room temperature with species-specific horseradish peroxidase (HRP)-conjugated secondary antibodies (Cell Signaling) for 1 h. Protein bands were visualized using an enhanced chemiluminescence (ECL) substrate (Pico PLUS, Thermo Fisher Scientific), and the signals were detected using a digital chemiluminescence imaging system (Bio-Rad Laboratories, Hercules, CA, USA). Band intensity was quantified using ImageJ software (National Institutes of Health, Bethesda, MD, USA), and values were normalized to β-actin.

### 2.4. Vitamin B_12_ ELISA

To confirm vitamin B deficiency, the concentration of vitamin B_12_ in serum samples from experimental mice was quantified using a commercial Enzyme-Linked Immunosorbent Assay (ELISA) kit (Aviva Systems Biology, San Diego, CA, USA, catalog # OKCA00147), according to the manufacturer’s protocol. Briefly, blood samples were collected from mice and allowed to clot at room temperature for 30 min. The samples were then centrifuged at 1000 rpm for 10 min at 4 °C to obtain clear serum, which was stored at −20 °C until analysis. For the assay, all reagents, standards, and samples were brought to room temperature before use. Standards of known vitamin B_12_ concentrations were prepared as specified in the kit instructions to generate a standard curve. A volume of 50 µL of each standard and diluted serum sample was added in duplicate to the wells pre-coated with anti-vitamin B_12_ antibodies. Then, 50 µL of the biotin-conjugated detection antibody was added to each well, followed by incubation at 37 °C for 1 h. 

After incubation, the wells were washed three times with 1× wash buffer to remove unbound materials. Subsequently, 100 µL of HRP-conjugated streptavidin solution was added to each well and incubated for 30 min at 37 °C. The wells were then washed again, and 90 µL of TMB (3,3′,5,5′-tetramethylbenzidine) substrate solution was added to develop color. The reaction was stopped after 15 min by adding 50 µL of stop solution, and the absorbance was immediately read at 450 nm using a microplate reader (BioTek Instruments, Winooski, VT, USA). Vitamin B_12_ concentrations in serum samples were calculated from the standard curve generated using known concentrations of the vitamin. The results were expressed as pg/mL of serum. All samples were analyzed in triplicate to ensure accuracy and reproducibility.

### 2.5. Homocysteine Assay (Fluorometric)

Following the establishment of B-vitamin deficiency, serum homocysteine concentrations were measured to confirm its associated elevation. The quantification of homocysteine was performed using a commercial Homocysteine Assay Kit (Abcam, Waltham, MA, USA, catalog # ab228559) according to the manufacturer’s instructions. After the collection of Blood samples, they were allowed to clot at room temperature for 30 min, then centrifuged at 1000 rpm for 10 min at 4 °C to obtain serum, and were stored at −20 °C until further biochemical analysis. Before the assay, all reagents, standards, and samples were equilibrated to room temperature. Standards with known concentrations of homocysteine were prepared to generate a standard calibration curve. For the fluorometric assay, 170 µL of each serum sample or standard was added to the designated wells of a 96-well microplate. The enzymatic reaction mixture provided in the kit, comprising homocysteine converting enzyme, cofactors, and substrate, was then added to each well according to the protocol.

The reaction mixture was incubated at 37 °C for 60 min. allowing enzymatic conversion of homocysteine to a detectable product. After incubation, fluorescence was measured using a microplate reader (BioTek Instruments, USA) at the appropriate wavelength. Homocysteine concentrations in the serum samples were calculated by interpolating the fluorescence values from the standard curve. The results were expressed as µmol/L of serum. All measurements were performed in triplicate to ensure accuracy and reproducibility.

### 2.6. Statistical Analysis

Statistical analyses were performed using GraphPad Prism software version 9 (GraphPad Software, La Jolla, CA, USA). Data are expressed as the mean ± standard deviation (SD). Differences between experimental groups were evaluated using either a two-tailed *t*-test or a one-way analysis of variance (ANOVA). When ANOVA indicated significant differences, Tukey’s post hoc test was applied to determine specific group comparisons. A *p*-value of less than 0.05 was considered statistically significant.

## 3. Results

### 3.1. Dietary B-Vitamin Manipulation Alters Serum Vitamin B_12_ and Homocysteine in Mice

**Figure 3:**
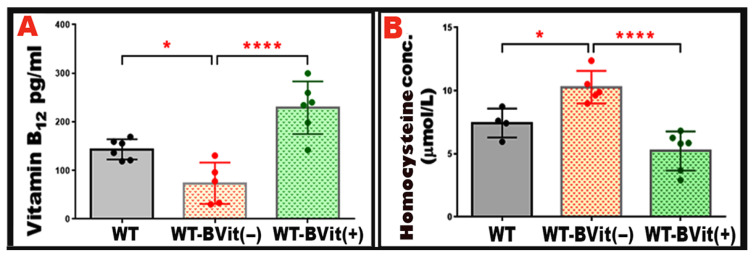
**Serum Vitamin B_12_ and Hcy levels**. Serum concentrations of vitamin B_12_ and Hcy were measured to assess B-vitamin status in the experimental groups, after 32 weeks of diet regimen initiation. (**A**) shows that WT mice fed a vitamin B-deficient diet (WT-B-Vit (−)) exhibited significantly reduced serum vitamin B_12_ levels, confirming the presence of B-vitamin deficiency. This deficiency was restored through B-vitamin supplementation, as demonstrated in the WT-B-Vit (+) groups. (**B**) The graph illustrates serum Hcy levels across the three groups (WT, WT-B-Vit (−), and WT-B-Vit (+)). It reveals that Hcy levels were significantly higher in the WT-B-Vit (−) group compared to the WT group. This elevation was notably reduced when shifting to a B-vitamin-enriched diet. Data are presented as mean ± SD, * *p* < 0.05, **** *p* < 0.001, n = 6.

To evaluate the effects of dietary changes in B-vitamins on systemic vitamin B_12_ levels and Hcy metabolism, serum vitamin B_12_ and Hcy levels were measured in the experimental groups: WT, WT-B-Vit (−), and WT-B-Vit (+). WT mice on the B-vitamin deficient diet showed a significant decrease in serum vitamin B_12_ concentrations compared to those on the regular diet (*p* = 0.0371). In contrast, the animals receiving the B-vitamin supplemented diet displayed elevated vitamin B_12_ levels when compared to WT that received the B-deficient diet, confirming the effectiveness of dietary modulation (*p* < 0.0001) (Figure 3A).

Serum Hcy concentrations were measured to evaluate the development of elevated Hcy levels. A significant increase in serum Hcy levels was observed in the mice fed the B-vitamin deficient diet (*p* = 0.0237 vs. WT). While supplementing with B-vitamins effectively normalized Hcy levels (*p* = 0.0002 vs. WT-BVit (−)) (Figure 3B), confirming that dietary enrichment reversed the elevated Hcy induced in our nutritional model. These findings demonstrate that deficiency of B-vitamins reliably elevates serum Hcy in mice, consistent with impaired one-carbon metabolism due to insufficient cofactors (vitamin B_12_, folate, B_6_). Conversely, dietary B-vitamin repletion restores B_12_ status and normalizes Hcy, confirming the reversibility of diet-induced metabolic disturbance. This supports the utility of dietary B-vitamin manipulation as a robust model for studying elevated Hcy level and its downstream effects (e.g., vascular, cognitive, epigenetic) in animal studies.

The concept of diet-induced modulation of Hcy via B-vitamin content is well established in the literature. For instance, a review of more than a hundred murine studies found negative correlations between dietary B_12_ and other one-carbon vitamins and circulating homocysteine levels [23].

### 3.2. Dietary B-Vitamin Deficiency Induces Retinal Vascular Leakage via Blood Retina Barrier Disruption in Mice, Which Is Attenuated by Vitamin B Supplementation

The BRB is crucial for maintaining retinal homeostasis and ensuring visual function. Impairment of the BRB, which leads to vascular leakage, is a key characteristic of retinal vascular diseases such as DR and AMD [12]. After confirming the establishment of our nutritionally induced Hcy model, we evaluated the BRB integrity. As previously reported in our studies, elevated Hcy disrupts both the inner and outer BRB integrity, both in vivo and in vitro. This disruption occurs through mechanisms that involve oxidative stress, inflammation, and the downregulation of tight-junction proteins [14]. In this context, we investigated whether a dietary deficiency of B-vitamins, which raises systemic Hcy levels, would compromise BRB integrity in the proposed nutritional mouse model. We conducted fundus fluorescein angiography (FA) in living mice to assess retinal vascular leakage under different dietary B-vitamin conditions: deficient, standard, and supplemented.

**Figure 4:**
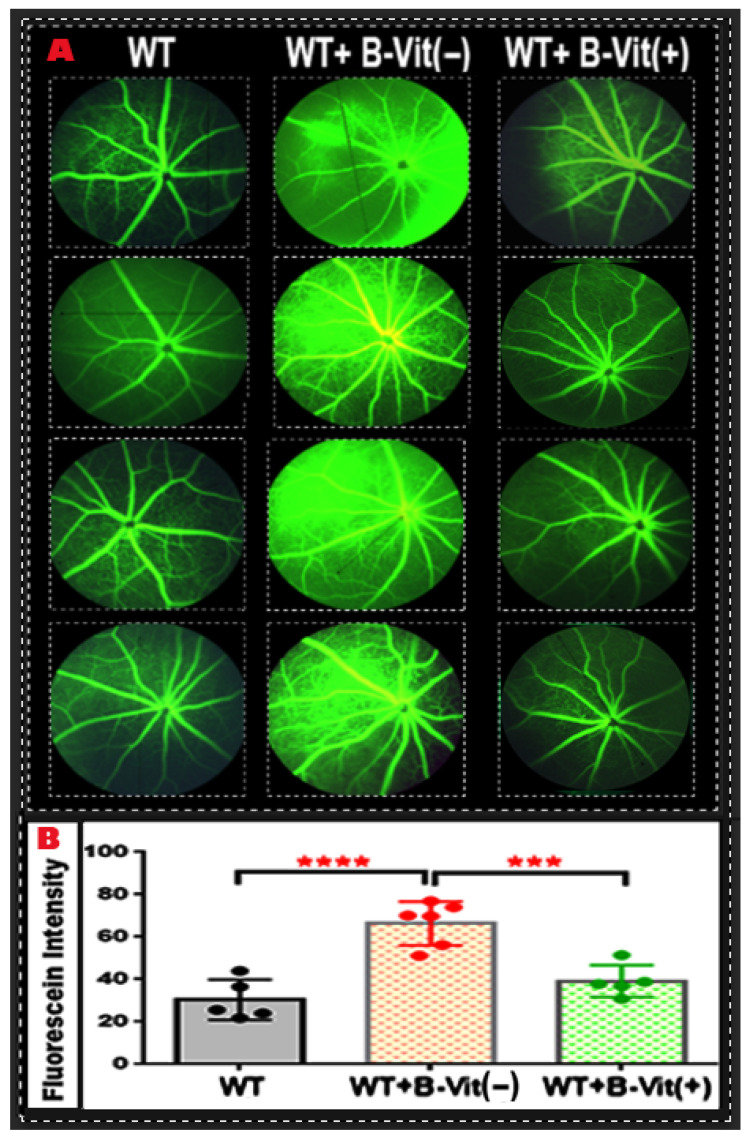
**Dietary B-Vitamin Deficiency Induces Retinal Vascular Leakage.** (**A**) Representative fundus FA images of mouse retinas, which were used to evaluate the integrity of the BRB. Mice fed the B-vitamin deficient diet (WT-B-Vit (−)) exhibited significant vascular leakage (areas of hyperfluorescence and dye leakage beyond the vessel borders) compared to the control WT mice. However, this leakage was markedly reduced following B-vitamin supplementation (WT-B-Vit (+)). The accompanying graph (**B**) displays the quantification of fluorescein intensity as measured using ImageJ. It reveals that mice on the B-vitamin deficient diet had a significantly higher fluorescein intensity compared to those on a regular diet. Conversely, vitamin supplementation effectively repaired the BRB and substantially decreased fluorescein leakage. Data are presented as mean ± SD, **** *p* < 0.0001, *** *p* = 0.0009, n = 6.

The FA images showed increased vascular leakage (indicated by diffuse hyperfluorescence, vessel wall staining, and leakage extending into the surrounding retinal tissue). in the retinas of mice on B-vitamin deficient diet (WT-B-Vit (−)) compared to those on a regular diet (*p* < 0.0001) (Figure 4), which was successfully mitigated by B-vitamin supplementation. Quantitative analysis of fluorescein intensity confirmed a significant increase in vascular leakage in the B-vitamin deficient group, which was notably reduced in mice that received a B-vitamin supplemented diet (WT-B-Vit (+)) (*p* = 0.0009 vs. WT-B-Vit (−)). These findings demonstrate that dietary B-vitamin deficiency compromises BRB integrity, resulting in vascular leakage in the retina, an effect that is reversed by B-vitamin supplementation. This strongly supports a mechanistic link between B-vitamin–dependent Hcy metabolism and retinal vascular homeostasis.

### 3.3. Dietary B-Vitamin Deficiency Induces Structural Retinal Alterations and Choroidal Neovascular Changes, Which Are Prevented by Vitamin B Supplementation

**Figure 5:**
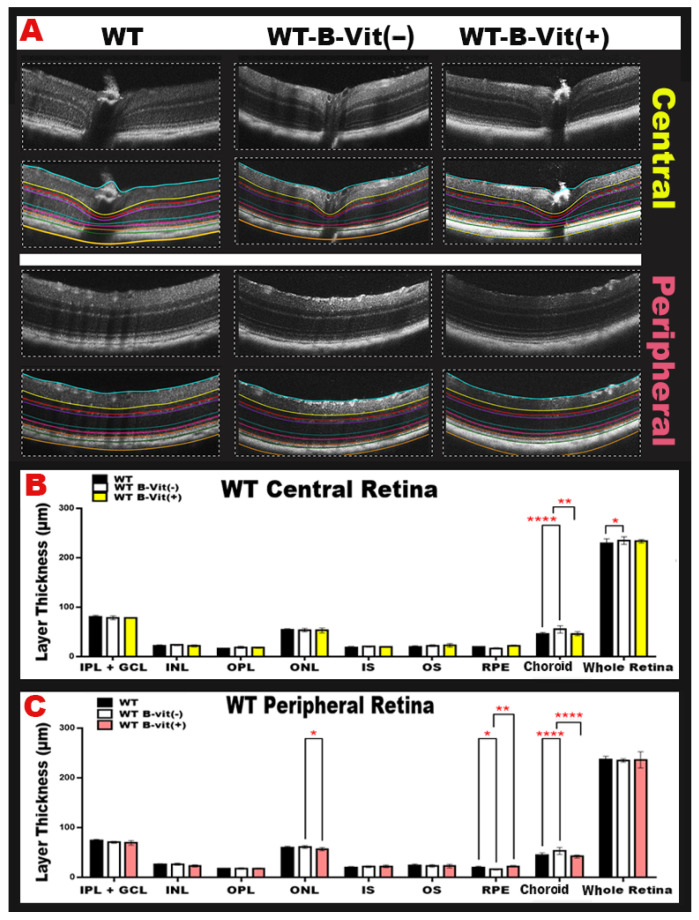
(**A**) Representative OCT images from each WT group under the customized B-vitamin regimen (WT, WT-B-Vit (−), and WT-B-Vit (+)) showing central and peripheral retinal structural changes, colored lines indicate the regions used for retinal thickness analysis. (**B**,**C**) The graphical representations of retinal thickness quantification for both central and peripheral retina reveal significant alterations primarily in the RPE/choroid. B-vitamin deficiency caused increased choroidal thickness (suggestive of choroidal neovascularization) and reduced RPE thickness, both of which were restored by B-vitamin supplementation. (IPL/GCL, inner plexiform layer/ganglion cell layer, INL inner nuclear layer, OPL outer plexiform layer, ONL outer nuclear layer, IS inner segment, OS outer segment, RPE retinal pigment epithelium). Data are presented as mean ± SD, * *p* < 0.05, ** *p* < 0.001, **** *p* < 0.0001, n = 24.

To determine how B-vitamin status affects retinal morphology, we performed optical coherence tomography (OCT) imaging across diet-modified WT groups. OCT imaging revealed structural changes in the retinas of mice deficient in B-vitamins. These changes included alterations in retinal thickness, localized inflammation, and areas of neovascularization (Figure 5A). Quantitative analysis of OCT images (Figure 5B,C). reflected these findings by showing significant changes in the thickness of different retinal layers in the B-vitamin deficient group compared to the WT controls (mainly in the RPE/choroid). A deficiency in B vitamins significantly increased choroidal thickness, indicating the presence of neovascularization, when compared to a regular diet group (*p* < 0.0001, peripheral and central retina). However, this condition was restored to normal with B-vitamin supplementation in both the peripheral (*p* < 0.0001) and central retina (*p* = 0.0026). Additionally, B vitamin deficiency led to a significant decrease in RPE thickness, suggesting cell death, compared to the regular diet (*p* = 0.0339). This thickness was also restored to normal levels with a diet rich in B-vitamins, particularly in the peripheral retina (*p* = 0.0026) (Figure 5C). Furthermore, B-vitamin supplementation protected the retina from the damaging effects of elevated Hcy, which is induced by B-vitamin deficiency, by restoring RPE thickness and reducing choroidal neovascularization. Collectively, these findings indicate that dietary B-vitamin deficiency drives structural retinal damage, including RPE loss and choroidal neovascular remodeling, while B-vitamin supplementation protects the retina by normalizing layer thickness and mitigating Hcy-induced injury.

### 3.4. B-Vitamin Deficiency Disrupts Tight Junction Integrity and Increases Retinal Albumin Leakage Through Homocysteine-Mediated BRB Breakdown

The BRB depends on tight junction proteins such as occludin and ZO-1 to maintain vascular integrity and prevent the extravasation of serum proteins into retinal tissue. Elevated Hcy, which arises from impaired one-carbon metabolism during B-vitamin deficiency, has been shown to destabilize tight junctions, increase endothelial permeability, and promote retinal barrier breakdown [14]. Given these established effects of elevated Hcy, we wanted to determine whether dietary B-vitamin deficiency disrupts BRB molecular components and whether B-vitamin supplementation can reverse these changes.

**Figure 6:**
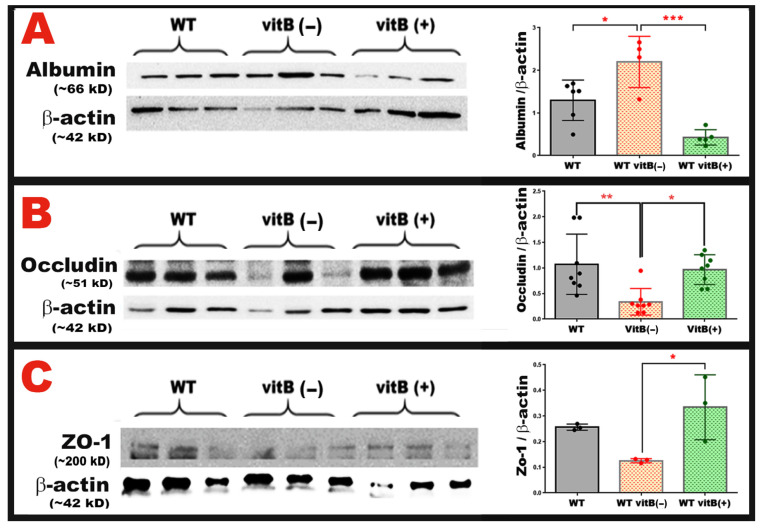
(**A**) Representative WB and quantitative analysis of retinal albumin levels in WT, WT-B-Vit (−), and WT-B-Vit (+). It reveals a significant increase in albumin levels in mice fed B-vitamin deficient diet (WT-B-Vit (−)) compared to WT controls. This elevation was attenuated by B-vitamin supplementation in the WT-B-Vit (+) group. (**B**,**C**) WB analyses of the tight junction proteins, occludin (**B**) and ZO-1 (**C**), in retinal tissue show a significant reduction in occludin expression in the WT-B-Vit (−) group compared to the WT group. Similarly, the ZO-1 levels were also reduced. Both protein expressions were restored in the B-vitamin supplemented group (WT-B-Vit (+)), as indicated in the accompanying graphs of their densitometric quantifications. Densitometry values were normalized to β-actin. Data are presented as mean ± SD, * *p* < 0.05, ** *p* < 0.005, *** *p* = 0.0002, n = 6.

Consistent with the angiographic and OCT findings, we investigated the molecular mechanisms underlying BRB dysfunction caused by B-vitamin deficiency and elevated Hcy. Following euthanasia and perfusion, retinas were collected for Western blot analysis (WB) of albumin accumulation and the tight junction proteins occludin and ZO-1 (Figure 6). As shown in Figure 6A, leaked retinal albumin levels were significantly elevated in mice fed the B-vitamin deficient diet (*p* = 0.0210 vs. WT), indicating increased vascular permeability and BRB breakdown. However, this albumin accumulation was markedly reduced in mice receiving B-vitamin supplementation (*p* = 0.0002 vs. B-Vit (−)), demonstrating a restoration of barrier integrity.

Analysis of tight junction protein expression revealed that both occludin and ZO-1 were significantly decreased in the B-vitamin deficient group compared with WT controls (Figure 6B,C) (*p* = 0.0046). B-vitamin supplementation effectively restored the expression levels of both proteins to near-normal levels (*p* = 0.0147, occludin; *p* = 0.0307, ZO-1). Together, these findings show that dietary B-vitamin deficiency compromises BRB integrity by reducing tight junction protein expression and increasing vascular leakage. Conversely, dietary B-vitamin supplementation mitigates these pathological effects, restoring tight junction organization and maintaining the structural and functional integrity of the BRB.

### 3.5. Nutritional B-Vitamin Deficiency Elevates Homocysteine to Levels Comparable to the Genetic cbs^+/−^ Model

Elevated Hcy levels can be induced through genetic or nutritional disruption of homocysteine metabolism. The heterozygous cystathionine β-synthase deficient mouse (*cbs^+/−^*) is a well-established genetic model of moderately elevated Hcy and has been extensively used to study the vascular and neurodegenerative consequences of elevated Hcy [21]. However, dietary B-vitamin deficiency provides a non-genetic approach that elevates Hcy by impairing the remethylation and transsulfuration pathways. To validate the strength of our nutritionally elevated Hcy model, we directly compared plasma Hcy levels across both models.

**Figure 7:**
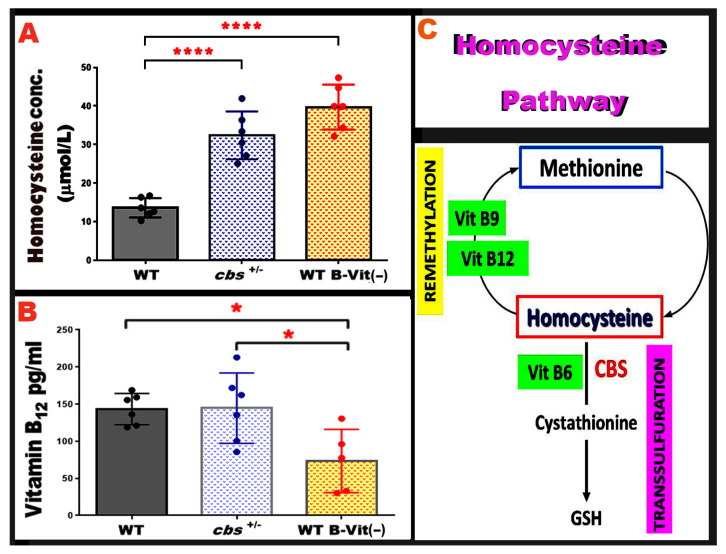
**Serum Hcy and vitamin B_12_ levels in both genetic and nutritional elevated Hcy models.** (**A**) The graph shows serum Hcy levels in WT, *cbs^+/−^*, and WT mice fed a B-vitamin deficient diet (WT-B-Vit (−)). The nutritional model, WT-B-Vit (−) produces a significant elevation in Hcy comparable to that observed in the genetic model *cbs^+/−^* compared to the WT. (**B**) Graph displays the serum vitamin B_12_ levels measured in WT, *cbs^+/−^*, and WT-B-Vit (−) groups, with a significant decrease in WT-B-Vit (−) as compared to WT control and the genetic model of elevated Hcy, *cbs^+/−^*. (**C**) Simplified Schematic of the Hcy metabolic pathway illustrating the role of vitamins B_6_, B_9_, and B_12_ in the remethylation and transsulfuration cycles. Data are presented as mean ± SD, * *p* < 0.05, **** *p* < 0.0001, n = 6.

Hcy concentrations were contrasted in mice fed the B-vitamin deficient diet with age- and sex-matched genetically induced elevated Hcy models (*cbs^+/−^*). As expected, *cbs^+/−^* mice showed significantly elevated Hcy levels relative to WT controls (*p* < 0.0001), consistent with previous reports. Remarkably, mice on the B-vitamin deficient diet exhibited a comparable magnitude of Hcy elevation (*p* < 0.0001), demonstrating that nutritional restriction of B-vitamins is equally effective in inducing elevated Hcy (Figure 7A). Furthermore, serum vitamin B_12_ level was compared across both models, and results showed that it is markedly lowered in the nutritionally induced model when compared to WT (*p* = 0.0245) and *cbs^+/−^* (*p* = 0.0218), as shown in Figure 7B. Together, these data confirm that the dietary B-vitamin deficiency model effectively induces elevated Hcy to levels similar to the genetic *cbs^+/−^* model.

### 3.6. B-Vitamin Status Modulates Retinal Vascular Leakage in Genetic and Nutritional Models of Elevated Homocysteine

**Figure 8:**
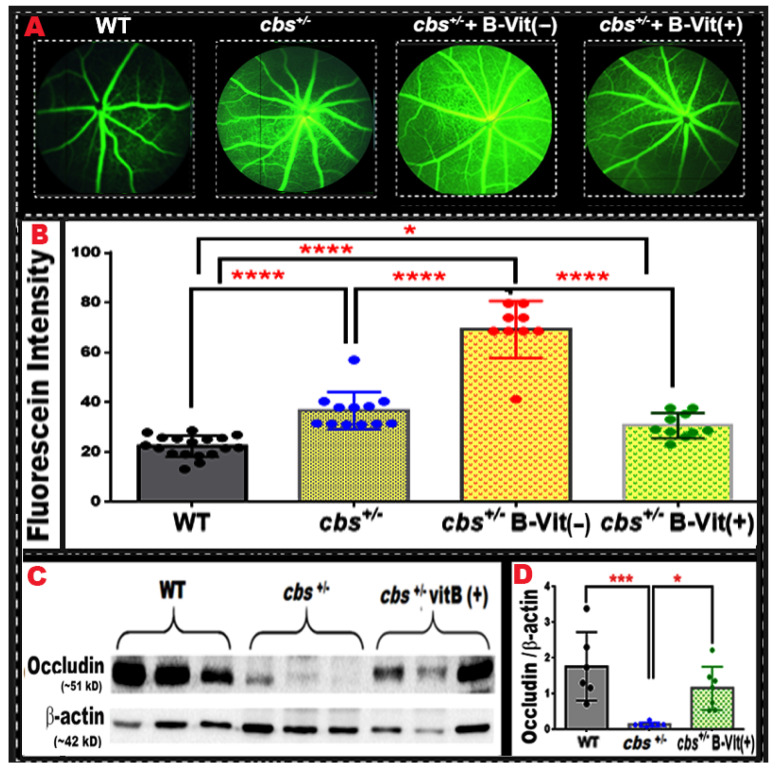
**Retinal FA reveals vascular leakage across genotypic and nutritional elevated Hcy models and the protective effect of B-vitamin supplementation.** (**A**) representative FA images from four groups: WT, *cbs^+/−^*, *cbs^+/−^* on B-vitamin–deficient diet (*cbs^+/−^* B-Vit (−)), and *cbs^+/−^* on B-vitamin–supplemented diet (*cbs^+/−^* B-Vit (+)). (**B**) The graph depicts the quantitative FA fluorescence intensity for each group. Compared with WT, *cbs^+/−^* mice displayed significantly increased fluorescence, indicating vascular leakage. This leakage was further elevated in *cbs^+/−^* B-Vit (−) mice, demonstrating an additive detrimental effect of B-vitamin deficiency on vascular integrity. B-vitamin supplementation in *cbs^+/−^* B-Vit (+) restored fluorescence levels toward baseline, indicating recovery of barrier function. (**C**) WB analysis of the tight junction protein, occludin, showing its significant reduction in *cbs^+/−^* group relative to WT and its restoration by B-vitamin supplementation. (**D**) Quantitative densitometric analysis of occludin WB band intensity normalized to the β-Actin. Data are presented as mean ± SD, * *p* < 0.05, *** *p* < 0.001, **** *p* < 0.0001, n = 6.

After establishing that dietary B-vitamin deficiency in WT mice causes retinal vascular and structural changes, we extended our analysis to the genetic model with Hcy elevation (*cbs^+/−^* mice) and tested how B-vitamin deficiency or supplementation affects retinal and BRB integrity. FA evaluation (Figure 8) showed low baseline fluorescence in WT mice, consistent with an intact BRB. In contrast, *cbs^+/−^* mice exhibited a significant increase in fluorescent leakage compared with WT controls, consistent with our previous reports demonstrating compromised vascular integrity in this genetic model of elevated homocysteine. Interestingly, when *cbs^+/−^* mice were placed on the B-vitamin–deficient diet (*cbs^+/−^* B-Vit (−)), we observed an even greater increase in FA leakage compared to both *cbs^+/−^* and WT, indicating synergistic worsening of vascular barrier breakdown when genetically elevated Hcy and nutritional deficiency are combined. Conversely, *cbs^+/−^* mice receiving B-vitamin supplementation (*cbs^+/−^* B-Vit (+)) showed a marked reduction in FA leakage, with fluorescence levels approaching those of WT, suggesting a restorative effect of B-vitamin supplementation on the BRB by acceleration Hcy metabolism.

These data support that B-vitamin status modulates the severity of elevated Hcy-induced retinal vascular leakage in both genetic and nutritional models. Consistent with these findings, Western blot analysis of the tight junction protein occludin (Figure 8C,D), revealed a significant reduction in occludin expression in *cbs^+/−^* retinas compared with WT (*p* = 0.0204), which was restored toward WT level by B-vitamin supplementation (*p* = 0.0498 vs. *cbs^+/−^)*, supporting a structural basis for the observed changes in vascular permeability.

Together with our results in WT mice fed a B-vitamin deficient diet, these findings demonstrate that the nutritional model of elevated Hcy can replicate vascular barrier dysfunction comparable to that seen in the genetic model. Moreover, B-vitamin repletion effectively ameliorates vascular leakage in *cbs^+/−^* mice.

To better understand our findings in a clinical context, elevated levels of plasma Hcy due to folate deficiency have been consistently linked to retinal vascular diseases in humans, including retinopathy and macular degeneration [24]. A cross-sectional study involving patients with type 2 diabetes revealed that higher total plasma Hcy levels were associated with an increased likelihood of proliferative retinopathy [25]. Furthermore, elevated Hcy levels have been connected to the thinning of the retinal nerve fiber layer, which serves as a marker for retinal neurovascular damage in diabetic patients [26].

### 3.7. B-Vitamin Supplementation Mitigates Retinal Structural Damage in cbs^+/−^ Mice

**Figure 9:**
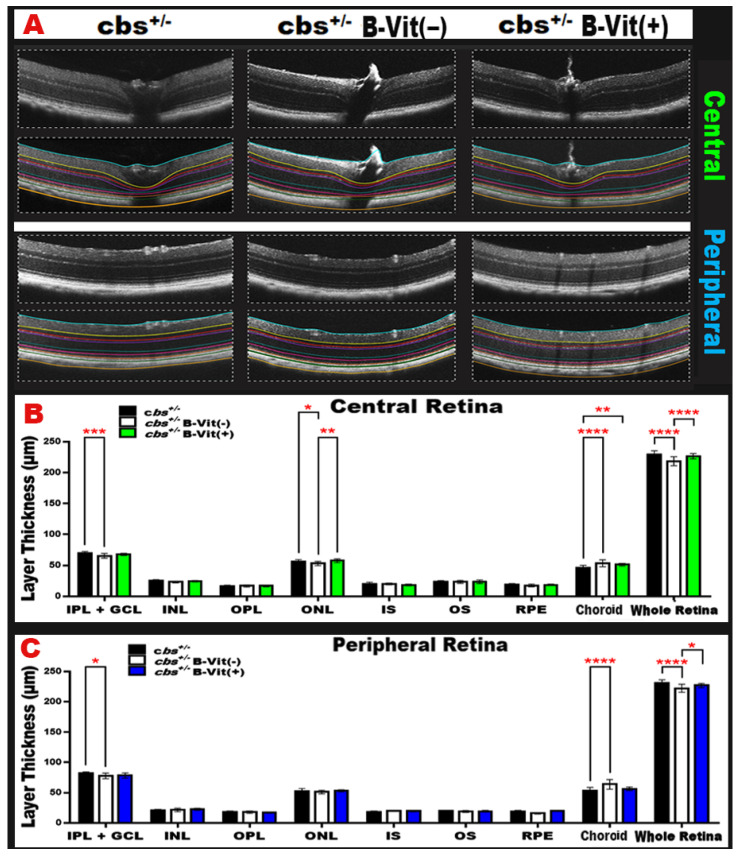
**Optical coherence tomography (OCT) imaging and retinal layer quantification in *cbs^+/−^* mice under different dietary regimens.** (**A**) Representative OCT images from *cbs^+/−^*, *cbs^+/−^*B-Vit (−), and *cbs^+/−^*B-Vit (+) groups, colored lines indicate the regions used for retinal thickness analysis. (**B**,**C**) represent graphs of retinal thickness quantification of central and peripheral retina, respectively, displaying significant effects in IPL + GCL, choroid, and total retina centrally, and in choroid, ONL, and IPL + GCL peripherally. B-vitamin deficiency increased choroidal thickness and reduced whole retinal thickness when compared to *cbs^+/−^* group on the regular diet, both corrected by B-vitamin supplementation. Supplementation also restored IPL + GCL and ONL thickness in deficient mice and mitigated elevated Hcy-induced neuronal loss (GCL, ONL), and choroidal neovascularization, improving overall retinal thickness. (IPL/GCL, inner plexiform layer/ganglion cell layer, INL inner nuclear layer, OPL outer plexiform layer, ONL outer nuclear layer, IS inner segment, OS outer segment, RPE retinal pigment epithelium). Data are presented as mean ± SD, * *p* < 0.05, ** *p* < 0.01, *** *p* < 0.001, **** *p* < 0.0001, n = 24.

Continuing to assess the retinal changes induced by B-vitamin deficiency in *cbs^+/−^* mice, OCT analysis (Figure 9A) was performed. Retinal layer quantification revealed significant effects of dietary manipulation on multiple retinal sublayers both centrally and peripherally. Analysis for the *cbs^+/−^* mice retina on our customized diet regimen (Figure 9B,C) revealed statistically significant changes in the IPL + GCL, choroid, and retina centrally, and choroid, ONL, and IPL + GCL peripherally. Mice with B vitamin deficient diet had an increase in choroidal thickness and a reduction in retinal thickness both centrally (*p* < 0.0001) and peripherally (*p* < 0.0001), compared to those that were on the regular diet, and this was corrected by B-vitamin supplementation. B-vitamin supplementation corrected the decrease in the IPL + GCL and ONL thickness in the B-vitamin deficient diet group peripherally (*p* < 0.0001 vs. *cbs^+/−^*B-Vit (−)). Moreover, B vitamin supplementation helped to reduce the effect of elevated Hcy-induced reduction in the neuronal part of the retina (GCL and ONL), choroidal neovascularization, and increased overall retinal thickness.

These findings suggest that B-vitamin deficiency exacerbates the elevated Hcy-induced retinal and choroidal structural changes, leading to neuronal loss and vascular alterations. Supplementation with B-vitamins appears to protect retinal neurons and maintain retinal and choroidal integrity, highlighting a potential therapeutic role for B-vitamin support in preventing elevated Hcy-mediated retinal degeneration.

## 4. Discussion

In this study, we implemented a long-term dietary approach using both C57BL/6 WT and *cbs^+/-^* mice to examine varying levels of B-vitamins intake (B_6_, B_9_, B_12_) over a period of 32 weeks. We confirmed vitamin B_12_ deficiency using ELISA and subsequently measured serum Hcy levels and retinal vascular endpoints. Our findings revealed that: (1) a dietary deficiency of B-vitamins consistently led to elevated serum Hcy levels, and that supplementation with B-vitamins reversed this elevation; (2) elevated Hcy was linked to increased retinal vascular leakage, as assessed by FA and OCT, along with structural changes in the retina; and (3) vitamin B supplementation alleviated indicators of BRB dysfunction, such as elevated retinal albumin levels and decreased expression of the tight junction proteins, occludin and ZO-1. These results support the notion that one-carbon metabolism, facilitated by B-vitamins, plays a critical role in maintaining the integrity of the retinal vascular barrier. Additionally, nutritional adjustments of B-vitamins and Hcy levels can influence the function of the blood–retinal barrier.

Our results are consistent with previous studies indicating that elevated Hcy contributes to microvascular dysfunction [27,28,29,30,31,32], and tight junction disruption [33,34]. Furthermore, genetic animal models of elevated Hcy, such as those deficient in the CBS enzyme or knockout models, show retinal vascular leakage and disruption of the blood–retinal barrier [35]. Our findings build upon this research by demonstrating that a dietary deficiency in B-vitamins produces similar outcomes. Additionally, we found that enriching the diet with B-vitamins can largely reverse these effects, thereby linking diet-driven one-carbon metabolism to the integrity of retinal microvasculature.

In our previous studies, we reported that the BRB is dysfunctional in *cbs^+/−^* mice. We observed harmful effects of elevated Hcy levels on the integrity of both the inner and outer BRB. These effects were noted both in vivo, using *cbs^+/−^* and *cbs^-/-^* mice, and in vitro, using cultured retinal cells [17,36]. Additionally, we explored different possible underlying mechanisms that disrupt the BRB, including oxidative stress, ER stress, inflammation, epigenic modification, activation of NMDAR, and retinal cellular metabolic dysfunction [15,18,19,20,21]. Our current study confirms these mechanisms through observed decreases in tight junction protein levels and increased albumin leakage.

The dietary intervention demonstrates that the levels of vitamins B_6_, B_9_, and B_12_ are crucial factors influencing systemic Hcy levels, consistent with human studies showing that low levels of B-vitamins are associated with elevated Hcy and increased vascular risk [37], Clinical studies have shown similar results regarding the beneficial effects of B vitamins, especially folic acid, in maintaining vascular health and reducing the incidence of microvascular disorders, including AMD [38]. Notably, our supplementation group reveals that higher B-vitamin intake can lower Hcy levels and improve vascular changes in the retina, indicating that nutritional interventions may help prevent or reverse microvascular barrier damage linked to elevated Hcy.

Our results demonstrated increased vascular leakage on FA, along with altered retinal thickness and structural changes detected by OCT in the vitamin B deficient groups. These findings suggest that the resulting elevated Hcy is associated with retinal vascular dysfunction and structural alterations, and is likely involved in mechanisms underlying retinal vascular leakage and structural remodeling. These findings align with our previous reports of retinal ganglion cell loss, retinal thinning, and capillary dropout in models of elevated Hcy level [14]. The partial restoration of retinal thickness and the reduced leakage observed in the groups supplemented with B-vitamins suggest that some of these changes may be reversible when the underlying metabolic issue is addressed. Furthermore, the Western Blot results indicated an increase in albumin levels and a decrease in tight junction proteins in the retinas of B-vitamin-deficient mice, which normalizes with supplementation. This provides molecular evidence of a compromised barrier. The current data strongly suggest that the endothelial tight junction complex is a significant site of damage, consistent with previous research [21].

Retinal vascular leakage and BRB impairment are key factors in major retinal diseases like DR and AMD. Our data may be clinically relevant, as studies have linked elevated Hcy levels to retinal vascular diseases [14,15,21,39,40,41]. The current study indicates that ensuring adequate B-vitamin levels may serve as a preventive or supportive strategy for promoting retinal vascular health, especially in populations at risk of elevated Hcy, such as the elderly, individuals with renal disease, and those with vitamin B deficiencies. The finding that supplementation reduced damage in our model reinforces the idea that this is a nutritional risk factor that can be modified.

Disruption of the BRB is an early and central event in the pathogenesis of these retinal disorders, promoting vascular leakage, inflammation, and subsequent neurovascular damage. The current study centers on BRB integrity as a critical determinant of retinal health, given its central role in retinal aging diseases marked by early neurovascular injury that precedes and drives retinal degeneration, such as AMD and DR.

Our emphasis on BRB dysfunction is supported by our previous work demonstrating that elevated Hcy is associated with vascular abnormalities and barrier breakdown in AMD, including neovascular AMD in human subjects [17]. In parallel, we reported increased Hcy levels in both human patients with DR and in experimental animal models, where Hcy elevation is linked to retinal vascular permeability, tight junction disruption, and inflammatory signaling [16]. In this study, we propose a safe nutritional model of elevated Hcy and BRB disruption, an important pathological feature shared across many retinal diseases, particularly those associated with aging. Aging populations are especially vulnerable to B-vitamin deficiency due to multiple factors, including inadequate dietary intake, malabsorption, drug interactions, and comorbid age-related conditions that impair B-vitamin absorption.

Together, these findings identify BRB dysfunction as a central targetable mechanism driving retinal aging and degeneration, rather than a secondary consequence of disease progression. By linking elevated Hcy, a hallmark of disrupted one-carbon metabolism to vascular dysfunction and BRB breakdown, our findings introduce a metabolically driven model in which barrier failure represents an early and modifiable event in retinal pathology.

This study also highlights the critical influence of nutritional status on BRB integrity and provides a solid structural foundation for future mechanistic and translational investigations. OCT-based retinal thickness measurements offer robust structural correlates of BRB disruption and enable the rational integration of complementary functional and inflammatory assessments in subsequent studies. Importantly, the consistent findings in *cbs^+/−^* mice underscore the pathogenic relevance of elevated Hcy, setting the stage for targeted Hcy-modulating interventions and validation across additional disease models, ultimately highlighting BRB dysfunction as a therapeutic target in retinal aging and degenerative diseases.

## 5. Conclusions

In conclusion, our data provide considerable experimental evidence that a dietary deficiency of vitamins B_6_, B_9_, and B_12_ can lead to higher Hcy levels, which subsequently cause disruptions in the BRB and increase retinal vascular leakage in mice. However, supplementation with B-vitamins can reverse these harmful changes. These findings underscore the critical role of one-carbon metabolism and adequate B-vitamin availability in maintaining retinal vascular health and suggest that nutritional interventions may represent a practical strategy to preserve BRB integrity.

## Figures and Tables

**Table 1:** Study design and biological N per endpoint.

Experimental Groups	WT-Control	WT-B-Vit (−)	WT-B-Vit (+)	*cbs^+/−^*	*cbs^+/−^*-B-Vit (−)	*cbs^+/−^*-B-Vit (+)
**Diet**	Regular	B-vitamin deficient	B-vitamin supplemented	Regular	B-vitamin deficient	B-vitamin supplemented
**Animals/group (n)**	8–10	8–10	8–10	8–10	8–10	8–10
**Initiation of diet regimen**	Baseline	Baseline	16 weeks	Baseline	Baseline	16 weeks
**OCT, FA**	Every 4 weeks	Every 4 weeks	Every 4 weeks	Every 4 weeks	Every 4 weeks	Every 4 weeks
**Hcy, vit B_12_ ELISA**	16 weeks 32 weeks	16 weeks 32 weeks	32 weeks	16 weeks 32 weeks	16 weeks 32 weeks	32 weeks
**Euthanasia and sample collection**	32 weeks	32 weeks	32 weeks	32 weeks	32 weeks	32 weeks
**Exclusions/Attrition**	Age- and sex-matched mice were used; old mice, pups, and pregnant females were excluded/Very sick and diabetic mice were not used to specifically analyze the effect of a specially formulated diet on BRB integrity.					
**Values of n**						
Serum Hcy and vit-B_12_ assays	n = 6	n = 6	n = 6	n = 6	n = 6	n = 6
Fluorescein intensity	n = 6	n = 6	n = 6	n = 6	n = 6	n = 6
OCT analysis	n = 24	n = 24	n = 24	n = 24	n = 24	n = 24
**WB analysis**						
Albumin	n = 6	n = 6	n = 6			
ZO-1	n = 3	n = 3	n = 3			
Occludin	n = 6	n = 6	n = 6	n = 6		n = 6

The entire experimental procedure, including dietary interventions, monitoring, and group transitions, was carried out in triplicate to ensure reproducibility.

## Data Availability

The data presented in this study are available on request from the corresponding author due to privacy.

## Abbreviations

The following abbreviations are used in this manuscript:

AMD	Age-related Macular Degeneration
BRB	Blood retinal barrier
CBS	Cystathionine β-synthase
DR	Diabetic Retinopathy
ELISA	Enzyme-Linked Immunosorbent Assay
FA	Fluorescein angiography
GCL	Ganglion cell layer
Hcy	Homocysteine
INL	Inner nuclear layer
IPL	Inner plexiform layer
IS	Inner segment
NMDARs	N-methyl-D-aspartate receptors
OCT	Optical coherence tomography
ONL	Outer nuclear layer
OPL	Outer plexiform layer
OS	Outer segment
RPE	Retinal pigment epithelium
WB	Western Blot analysis
WT	Wild-type
